# Investigating the causal association between obesity and risk of hepatocellular carcinoma and underlying mechanisms

**DOI:** 10.1038/s41598-024-66414-1

**Published:** 2024-07-08

**Authors:** Zhitao Chen, Chenchen Ding, Kailei Chen, Yangjun Gu, Xiaoxia Qiu, Qiyong Li

**Affiliations:** 1 Department of Hepatobiliary Surgery, Shulan (Hangzhou) Hospital Affiliated to Zhejiang Shuren University Shulan International Medical College, 848# Dongxin Road, Hangzhou, 310003 Zhejiang Province China; 2 Child and Adolescent Psychology, Affiliated Mental Health Centre & Hangzhou Seventh People’s Hospital, Zhejiang University School of Medicine, Hangzhou, 310013 Zhejiang China; 3 School of Medicine, Zhejiang Shuren University, Hangzhou, 310003 China

**Keywords:** Obesity, Hepatocellular carcinoma, Mendelian randomization, Body mass index, Body fat percentage, Immune cell infiltration, Cancer, Drug discovery, Oncology, Risk factors, Genetics

## Abstract

Obesity is a global health concern and independent risk factor for cancers including hepatocellular carcinoma (HCC). However, evidence on the causal links between obesity and HCC is limited and inconclusive. This study aimed to investigate the causal relationship between obesity-related traits and HCC risk and explore underlying mechanisms using bioinformatics approaches. Two-sample Mendelian randomization analysis was conducted leveraging publicly available genome-wide association study summary data on obesity traits (body mass index, body fat percentage, waist circumference, waist-to-hip ratio, visceral adipose tissue volume) and HCC. Associations of obesity with primary mechanisms (insulin resistance, adipokines, inflammation) and their effects on HCC were examined. Differentially expressed genes in obesity and HCC were identified and functional enrichment analyses were performed. Correlations with tumor microenvironment (TME) and immunotherapy markers were analyzed. Genetically predicted higher body mass index and body fat percentage showed significant causal relationships with increased HCC risk. Overall obesity also demonstrated causal links with insulin resistance, circulating leptin levels, C-reactive protein levels and risk of severe insulin resistant type 2 diabetes. Four differentially expressed genes (ESR1, GCDH, FAHD2A, DCXR) were common in obesity and HCC. Enrichment analyses indicated their roles in processes like RNA capping, viral transcription, IL-17 signaling and endocrine resistance. They exhibited negative correlations with immune cell infiltration and immunotherapy markers in HCC. Overall obesity likely has a causal effect on HCC risk in Europeans, possibly via influencing primary mechanisms. The identified differentially expressed genes may be implicated in obesity-induced hepatocarcinogenesis through regulating cell cycle, inflammation and immune evasion. Further research on precise mechanisms is warranted.

## Introduction

Hepatocellular carcinoma (HCC), a highly aggressive form of primary liver cancer, ranks as the seventh most prevalent malignant tumor globally, characterized by significant cancer-related mortality^1–4^. HCC exhibits an asymptomatic progression during its early stages and demonstrates metastatic potential in advanced stages, often resulting in a diagnosis at a terminal phase and associated with a poor prognosis^5,6^. Despite significant advancements in therapeutic strategies for HCC, such as targeted therapy and immunotherapy in recent decades, the relatively higher propensity for recurrence and metastasis compared to other malignancies has hindered substantial improvements in 5-year overall survival rates^5,7,8^. Due to the substantial healthcare and economic burden attributed to HCC, there has been a growing emphasis on HCC prevention in recent years within the academic and medical community^9^. Therefore, investigating the underlying risk factors and biological mechanisms driving HCC development not only aids in preventing the occurrence of HCC but also offers the potential for advancing comprehensive cancer therapies.

The etiology of HCC remains not entirely clear. Epidemiological investigations have implicated several risk factors in the development of HCC, including infections with Hepatitis B Virus and Hepatitis C Virus, as well as the chronic necroinflammatory liver damage induced by alcohol consumption or non-alcoholic fatty liver disease^1,10,11^. Obesity, often stemming from an imbalance between energy intake and expenditure, was officially categorized as an epidemic by the World Health Organization (WHO) in 1997 and is highly prevalent in both developed and developing nations^12^. Obesity, a rapidly escalating public health concern, is recognized as an independent risk factor for numerous types of cancer, particularly those associated with the digestive system^13–15^. Fat is deposited in various depots, including subcutaneous abdominal, gluteofemoral, and intra-abdominal (visceral) adipose tissue. These depots arise from distinct fat progenitors, which subsequently differentiate into specialized adipocytes exhibiting a range of functional characteristics^16,17^. The cellular and molecular foundations of the relationship between obesity and cancer are intricate and not fully elucidated. Currently, three primary mechanisms have been postulated to establish a link between obesity and cancer, encompassing insulin resistance, adipokines—most notably, leptin and adiponectin—and chronic inflammation^18–20^.

Nonetheless, the available evidence regarding the connection between obesity and HCC is limited and inconclusive, leaving the question of causality uncertain^13,21^. Investigating the causal links between obesity and HCC holds great significance in the understanding of predisposing factors for diseases and in informing clinical treatment decisions. Randomized controlled trials (RCTs) are considered the gold standard for determining causal relationships between diseases and interventions; however, they are often costly and time-consuming to conduct. The inherent impracticality of conducting RCTs to establish a causal relationship between obesity and HCC necessitates the need for Mendelian randomization (MR) analysis. The MR analysis, employing one or more genetic variants as instrumental variables (IVs) for the risk factors of concern, has found extensive application in elucidating causal relationships between exposures and outcomes, including human diseases and disease phenotypes. Currently, no researchers have undertaken an examination of the causal relationship between various obesity-related traits and HCC. The objective of this study is to utilize the MR causal framework to assess whether five obesity traits, namely body mass index (BMI), body fat percentage, waist circumference, waist-to-hip ratio, and visceral adipose tissue volume, have a causal impact on the development of HCC. We are also employing bioinformatics to comprehensively investigate the underlying pathogenic mechanisms of obesity-induced HCC and to guide its treatment.

## Materials and methods

### Study design

**Figure 1:**
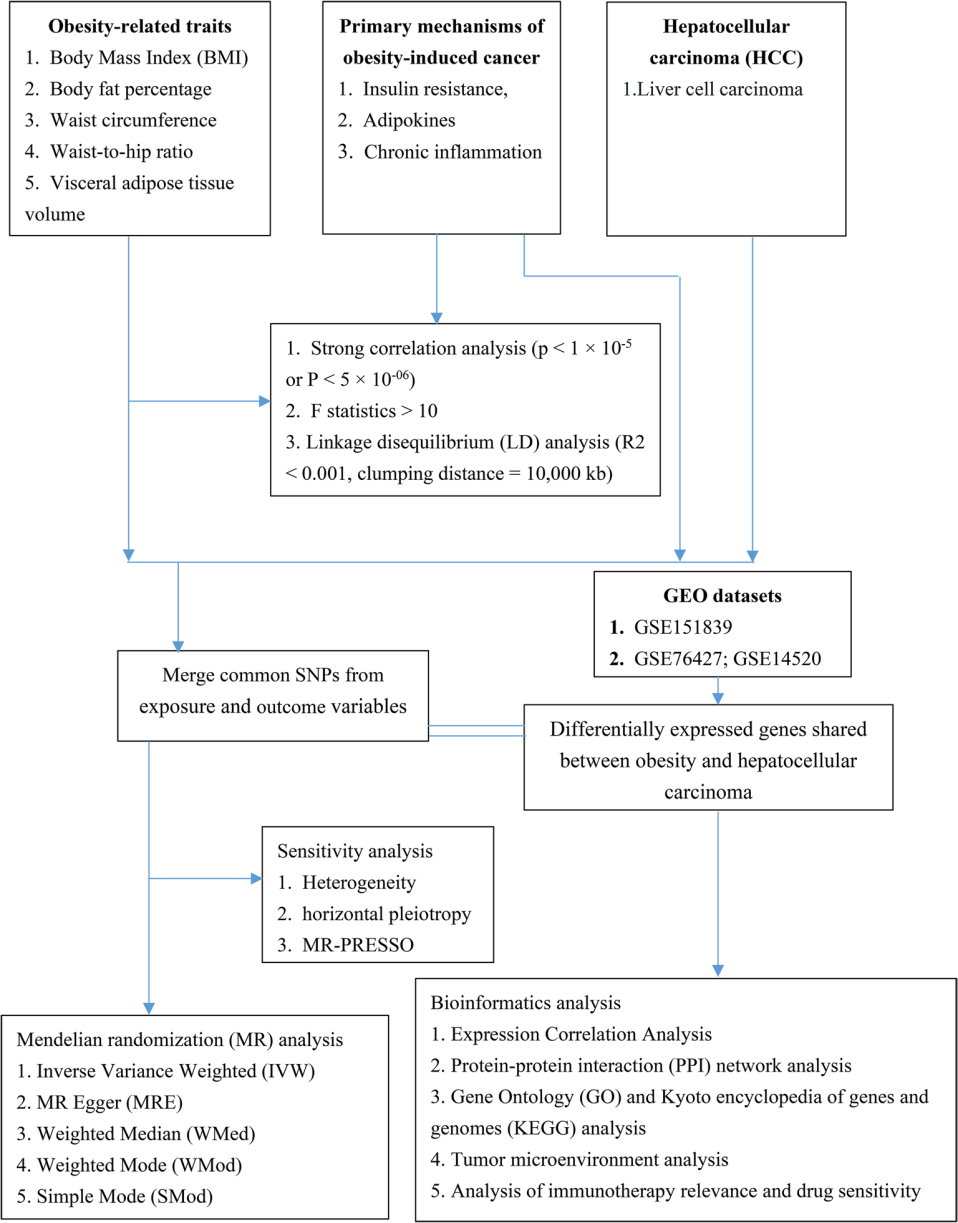
Flowchart of present study. *BMI* body mass index, *HCC* hepatocellular carcinoma, *LD* linkage disequilibrium, *SNPs* single-nucleotide polymorphisms, *GEO* Gene expression omnibus, *MR* Mendelian randomization, *IVW* inverse variance weighted, *MRE* MR egger, *WMed* weighted median, *WMod* weighted mode, *SMod* simple mode, *PPI* protein–protein interaction, *GO* gene ontology, *KEGG* Kyoto encyclopedia of genes and genomes.

This research utilized a two-sample MR approach, leveraging publicly accessible datasets for all analyses. Initially, MR was used to assess the causal impact of five obesity-related traits on HCC in a European population. This was followed by a detailed two-sample MR analysis aimed at exploring the connections between these obesity-related traits and the three primary mechanisms of obesity-induced cancer: insulin resistance, adipokines, and chronic inflammation. Additionally, we conducted an examination to explore the correlation between these mechanisms and the risk of HCC development in individuals of European descent. To investigate the causal relationship between obesity-related traits and HCC across different ethnic groups, we selected Genome Wide Association Study (GWAS) data from Asian populations for further analysis. Finally, we conducted a bioinformatics analysis of genes co-expressed in obesity and HCC using the Gene Expression Omnibus (GEO) database. This analysis aimed to explore potential pathogenic mechanisms and provide guidance for treatment. The process for each stage of the study is depicted in Fig. 1.

### Data sources

GWAS is a research methodology that investigates genetic factors linked to complex diseases through the analysis of extensive DNA samples from large populations, enabling a comprehensive exploration of genes implicated in the onset, progression, and therapeutic aspects of various illnesses. The exposure and outcome datasets for the current study were acquired from the OpenGWAS, GWAS catalog and BBJ database. To better distinguish the causal relationship between obesity and HCC across different features, we subdivided obesity-related traits. Within these GWAS datasets, BMI and body fat percentage serve as metrics for overall obesity, while waist circumference and waist-to-hip ratio reflect abdominal obesity. Additionally, visceral adipose tissue volume is used as an index for visceral obesity. The primary data related to the three primary mechanisms of obesity-induced cancer have also been explored. In particular, C-reactive protein levels were indicative of chronic inflammation indexes, while circulating leptin levels and adiponectin represented adipokines indexes. Additionally, severe insulin-resistant type 2 diabetes served as an indicator for insulin resistance indexes. For the GWAS dataset on HCC in the European population, we utilized summary-level data from the UK Biobank, which includes 168 cases, 372,016 controls, and 6,304,034 single-nucleotide polymorphisms (SNPs). Similarly, for the GWAS dataset on HCC in the Asian population, we used summary-level data from the BBJ database, comprising 2122 cases, 159,201 controls, and 13,433,488 SNPs. A summary of the detailed data is provided in Table 1.

**Table 1:** The genome-wide association studies (GWAS) datasets in present Mendelian randomization (MR) analysis.

GWAS ID	Traits	Author/Year	Consortium	Population	Sample size	SNPs
*Overall obesity indexes*						
ukb-a-248	Body mass index (BMI)	Neale/2017	Neale Lab	European	336,107	10,894,596
ukb-b-8909	Body fat percentage	Ben Elsworth/2018	MRC-IEU	European	454,633	9,851,867
*Abdominal obesity indexes*						
ukb-b-9405	Waist circumference	Ben Elsworth/2018	MRC-IEU	European	462,166	9,851,867
ieu-a-73	Waist-to-hip ratio	Shungin D/2015	GIANT	European	212,244	2,560,782
*Visceral adipose indexes*						
ebi-a-GCST90016671	Visceral adipose tissue volume	Liu Y/2021	NA	European	32,860	9,275,407
*Chronic inflammation indexes*						
ebi-a-GCST90029070	C-reactive protein levels	Said S/2022	NA	European	575,531	10,713,245
*Adipokines indexes*						
ebi-a-GCST90007307	circulating leptin levels	Yaghootkar H/2020	NA	European	56,802	231,001
ieu-a-1	Adiponectin	Dastani Z/2012	ADIPOGen	Mixed	39,883	2,675,209
*Insulin resistance indexes*						
ebi-a-GCST90026414	Severe insulin-resistant type 2 diabetes	Mansour Aly D/2021	NA	European	3,874	5,376,977
*Hepatocellular carcinoma*						
ieu-b-4953	Liver cell carcinoma	Burrows/2021	UK Biobank	European	372,184	6,304,034
*Asian population*						
HepC: Hepatic cancer	Hepatocellular carcinoma	Sakaue S/2021	BBJ	Asian	161,323	13,433,488
BMI: Body mass index	BMI	Sakaue S/2021	BBJ	Asian	163,853	162,364,65
GCST90103752	Body fat percentage	Wong HS/2022	GWAS catalog	Asian	21,304	6,546,461

*GWAS* genome-wide association studies, *MR* Mendelian randomization, *BMI* body mass index, *ID* Identification, *MRC-IEU* Medical Research Council Integrative Epidemiology Unit, *GIANT* Genetic Investigation of Anthropometric Traits, *ADIPOGen* Adiposity and Fat Distribution Genetics Consortium, *UK* United Kingdom, *NA* no available.

### Data screening

IVs were subjected to the following criteria: (1) SNPs meeting the locus-wide significance threshold of *P* < 5 × 10^−08^ were meticulously selected as prospective IVs associated with exposure factors. It is worth noting that when severe insulin-resistant type 2 diabetes (ebi-a-GCST90026414) and Body fat percentage (GCST90103752) were treated as an exposure, no corresponding IVs with SNPs meeting the locus-wide significance threshold of *P* < 5 × 10^−08^ were identified. Therefore, we relaxed the locus-wide significance threshold to *P* < 5 × 10^−06^ for selecting IVs for this exposure. (2) To obtain independent IVs from different loci, an analysis of the 1000 Genomes EUR/ESA dataset employed a linkage disequilibrium (LD) threshold of R^2^ < 0.001 and a clumping distance of 10,000 kb. (3) Summary data of the IVs related to the exposure indicator under investigation were extracted, and the F statistics (F = beta^2^/se^2^) were used to assess the strength of the IVs^22^. A value exceeding 10 was considered indicative of a robust instrument. These conditions were employed to identify more reliable IVs in this study, thereby enhancing the reliability of the study’s results.

### MR analysis and statistical analysis

RStudio (version 4.1.3) was utilized to conduct the MR analysis and statistical analysis in this study. The TwoSampleMR package (version 0.5.7, available at https://github.com/MRCIEU/TwoSampleMR) was utilized to integrate and harmonize data pertaining to obesity-associated traits, primary mechanisms of obesity-induced cancer, and HCC in two-sample MR analyses. The primary method employed for assessing the overall causal effect of the exposure on the outcome was the Inverse Variance Weighted (IVW) method, assuming a fixed-effect model. The IVW method aimed to minimize the weighted average variance by aggregating two or more IVs, with each IV’s weight being the reciprocal of the variance of the effect estimate. In addition to the IVW method, we conducted supplementary analyses to estimate causal effects using Weighted Median Regression (WMR), Egger regression (MR Egger) method, Weighted Mode (WMod), and Simple Mode (SMod) methods. The five statistical methods used in MR each cater to different scenarios of genetic instrument validity and data robustness. The IVW method assumes all genetic variants are valid instruments and computes a weighted average based on the inverse of each variant’s variance. In contrast, the MRE method allows for the inclusion of some invalid instruments by adjusting for horizontal pleiotropy through penalized regression. The WMed method offers robust estimates by focusing on the median ratio, effective even if up to 50% of the data comes from invalid instruments. The WMod method emphasizes the most frequent ratio estimate provided it originates from valid instruments. Lastly, the SMod method, similar to WMod but without weighting, picks the most commonly occurring ratio across all variants, regardless of their variance, offering a straightforward but less nuanced approach. In our causal estimation, a *P*-value of less than 0.05 was regarded as statistically significant.

### Sensitivity analysis

In the IVW model, heterogeneity is assessed using the Cochran’s Q test, and if *P* < 0.05, it is considered as evidence of heterogeneity. In such cases, a random-effects model would be selected. It is important to note that the effectiveness of the IVW model remains unaffected by the presence of heterogeneity. Horizontal pleiotropy was detected through MR-Egger intercepts. The MR-PRESSO method was applied to identify potential outliers. In the event of identifying outliers, the MR analyses were reperformed. The impact of an individual SNP on the results of the MR analysis was evaluated using a leave-one-out analysis. Scatter plots and funnel plots were generated to visually interpret the outcomes of the MR analyses and to identify any potential outliers within the data.

### Data sources and differential expression analysis of obesity and HCC samples

The GEO database (http://www.ncbi.nlm.nih.gov/geo) is a widely recognized and extensive repository of biological data pertaining to gene expression. In the present study, we obtained the GSE151839 dataset (Platforms: GPL570) from the GEO database, which includes 10 obese (BMI 35–50) and 10 normal weight (BMI 18.5–26.9) skin/fat samples. Additionally, GSE76427 (Platforms: GPL10558) and GSE14520 (Platforms: GPL571) are datasets that pertain to the study of HCC and normal liver tissues. Subsequently, we employed the GEO2R tool to extract differentially expressed mRNAs from the GSE76427 and GSE14520 datasets, applying a threshold of |log 2 (fold change [FC])|> 1 and *P*-value < 0.05 for selection. Due to the limited sample size in GSE151839, which hinders the identification of a significant number of differentially expressed mRNAs, we have decided to relax the criteria to include only those with a *P*-value < 0.05. Furthermore, we utilized the Venn diagram to identify common differentially expressed genes among these three datasets, which will be used for subsequent analyses.

### Protein–protein interaction (PPI), gene ontology (GO) and Kyoto encyclopedia of genes and genomes (KEGG) analysis

A PPI network for these differentially expressed genes was initially built using GeneMANIA (http://www.genemania.org), a valuable online tool for generating PPI networks, formulating hypotheses about gene functions, and identifying genes with similar functions. Additionally, the STRING website (https://string-db.org/) was employed to explore the binding proteins of these differentially expressed genes, and we also investigated 10 closely related genes. Lastly, a comprehensive analysis was conducted by merging the 4 differentially expressed genes with the 10 closely related genes. This integrated set was then subjected to GO enrichment analysis and KEGG pathway analysis using R packages, including clusterProfiler, org.Hs.eg.db, ggplot2, and enrichplot, all within the Rstudio. Statistical significance was determined at a threshold of *P* < 0.05.

### Tumor immune single-cell and immune cell infiltration analysis

The Tumor Immune Single-cell Hub (TISCH, http://tisch.comp-genomics.org/) is a comprehensive online database that houses single-cell RNA sequencing data. It offers a systematic investigation into the diversity of the tumor microenvironment (TME) across different datasets and cell types. In our analysis, we specifically utilized the LIHC_GSE140228_10X dataset from the TISCH database to examine the correlation between differentially expressed genes and the TME. The TIMER (https://cistrome.shinyapps.io/timer/) database was employed to investigate the association between differentially expressed genes and the levels of six categories of immune infiltrating cells, which include CD4 + T cells, CD8 + T cells, neutrophils, myeloid dendritic cells, macrophages, and B cells.

### Immunotherapy and drug sensitivity analysis

In order to investigate the response of HCC patients with comorbid obesity to immunotherapy, we conducted a search in the TISIDB (http://cis.hku.hk/TISIDB/index.php) database to establish the associations between differentially expressed genes and immune checkpoint inhibitors in HCC. Additionally, we employed scatter plots to illustrate the expression relationships between differentially expressed genes and PD-1 (PDCD1), PD-L1 (CD279), and CTLA4. We utilized the Genomics of Drug Sensitivity in Cancer (GDSC) website to assess a wide range of drugs in terms of their predictive capabilities for chemotherapy response. Differentially expressed genes were leveraged for predicting drug sensitivity, using data obtained from the GSCA (http://bioinfo.life.hust.edu.cn/) database. Simultaneously, the DrugBank (https://go.drugbank.com/) database was employed to investigate the chemical molecular formulas of sensitive drugs.

## Results

### Effect of obesity-related traits on HCC risk in the European population

Initially, we included a total of 315 index SNPs as IVs associated with BMI, 395 with body fat percentage, 374 with waist circumference, 29 with waist-to-hip ratio, and 5 with visceral adipose tissue volume, selected based on the predefined threshold for locus-wide statistical significance (*P* < 5 × 10^−8^) and the LD threshold (R^2^ < 0.001), with a clumping distance of 10,000 kb. The SNPs for each exposure demonstrated sufficient strength, with F-statistics exceeding 10 (29.75–997.38). Detailed information regarding the IVs for each exposure is provided in Table S1. Meanwhile, the integration of SNPs data from various obesity-related traits with HCC datasets is presented in Table S2.

**Figure 2:**
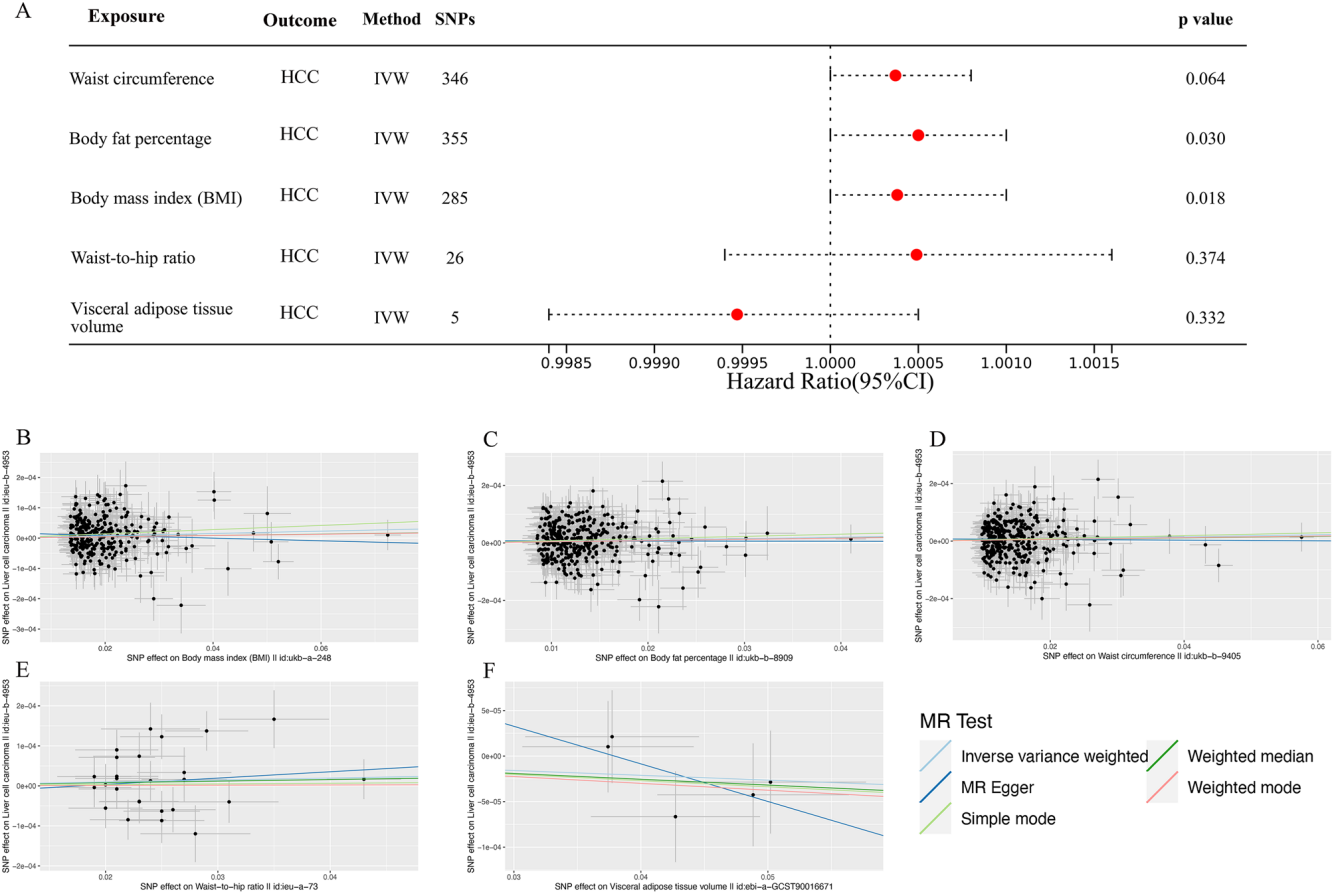
Associations of genetically predicted various obesity-related traits with risk of HCC using the IVW method. (**A**) A forest plot illustrates the connections between genetically predicted factors related to five obesity-related traits and the risk of developing HCC. (**B–F**) Scatter plots for the causal association between five obesity-related traits and HCC. *HCC* hepatocellular carcinoma, *IVW* inverse variance weighted.

A comprehensive two-sample MR analysis was systematically conducted to evaluate the potential causal relationship between obesity-related traits and the incidence of HCC. Using the IVW method, we observed that the genetic predisposition to increased BMI [*P* = 0.018, Odds ratio (OR) = 1.00038, 95% confidence interval (CI) 1.0001–1.0007] and body fat percentage (*P* = 0.030, OR = 1.00050, 95% CI 1.0000–1.0010) was associated with a higher risk of HCC (Table S3, Fig. 2A). The results obtained through the IVW method suggested that there was no significant causal relationship between waist circumference (*P* = 0.064, OR = 1.00037, 95% CI 1.0000–1.0008), waist-to-hip ratio (*P* = 0.374, OR = 1.00049, 95% CI 0.9994–1.0016), visceral adipose tissue volume (*P* = 0.332, OR = 0.99947, 95% CI 0.9984–1.0005), and HCC (Table S3, Fig. 2A). Scatter plots depicting the associations between genetically diverse obesity-related traits and the risk of HCC were presented in Fig. 2B–F.

### Effect of obesity-related traits on HCC risk in the Asian population

The comprehensive analysis presented here demonstrates that overall obesity (BMI and body fat percentage) is associated with HCC in the European population, as detailed in Table S3. Consequently, we have focused on extensively exploring the causal relationships between these obesity-related traits and HCC in the Asian population. Following the criteria for IV selection, 79 SNPs and 16 SNPs were identified as instrumental variables for BMI and body fat percentage, respectively, in the Asian population (Table S1). The results obtained through the IVW method suggested that there was no significant causal relationship between BMI (*P* = 0.349, OR = 1.16544, 95% CI 0.8462–1.6052), body fat percentage (*P* = 0.074, OR = 1.07728, 95% CI 0.7558–1.5355) and HCC (Table S3). Despite the *P*-values not meeting the threshold for statistical significance, the direction of the effect sizes (OR > 1) is consistent with the findings from our European cohort analyses. This consistency supports the hypothesis that obesity-related traits are potential risk factors for HCC, reinforcing the need for further investigation with larger datasets that could provide the power necessary to achieve statistical significance.

### Effect of overall obesity on three primary mechanisms of obesity-induced cancer

Based on the previous analysis, we have identified a causal relationship between systemic overall obesity-related traits and the risk of HCC. Therefore, this study primarily investigates the relationship between systemic overall obesity-related traits (BMI and body fat percentage) and the primary mechanisms of obesity-induced cancer. Firstly, detailed information regarding the IVs for BMI and body fat percentage is presented in Table S1. The amalgamation of SNPs data from BMI and body fat percentage with primary mechanisms of obesity-induced cancer datasets is depicted in Table S4.

**Figure 3:**
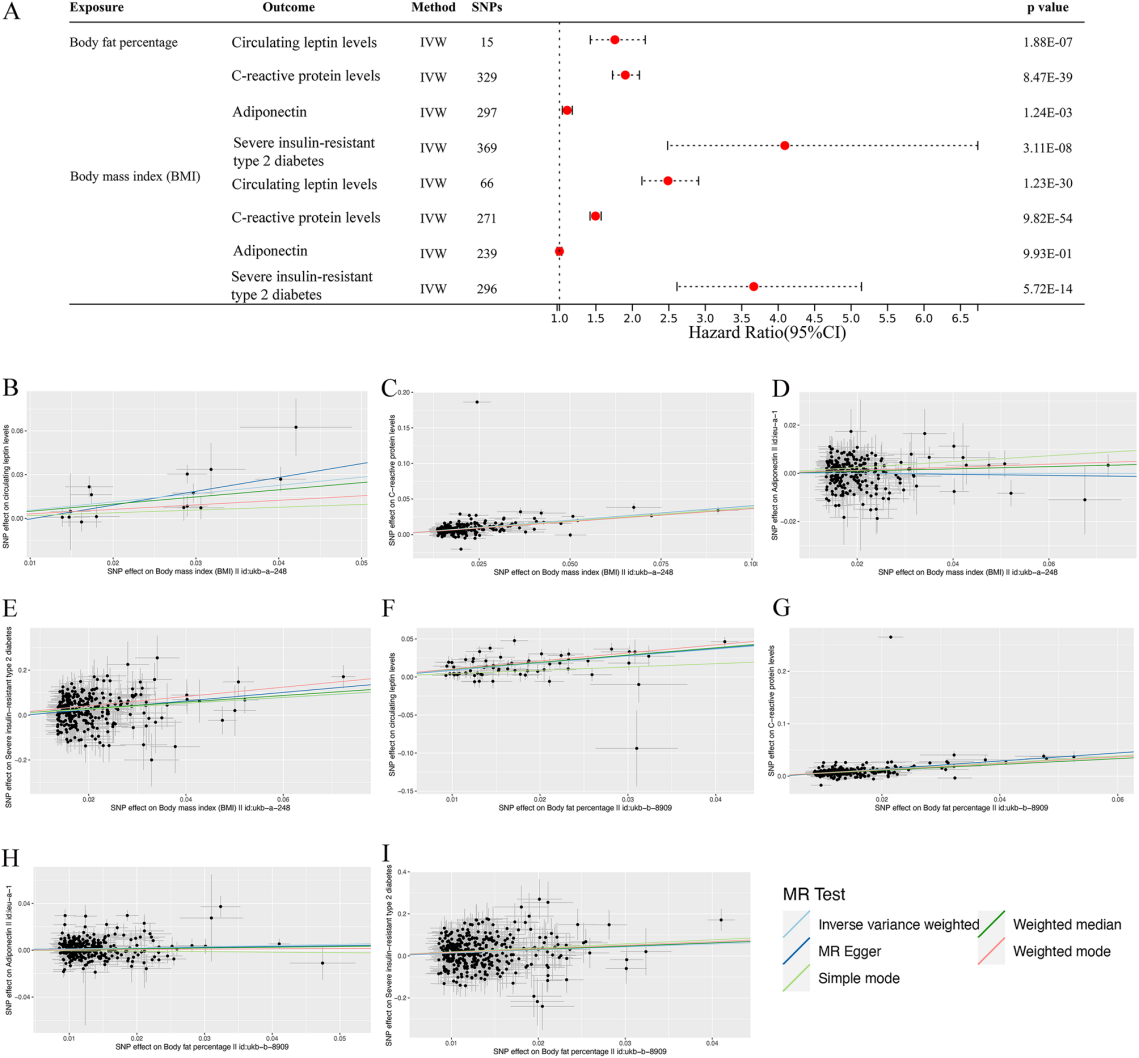
Associations of genetically predicted overall obesity (BMI and body fat percentage) with risk of primary mechanisms of obesity-induced cancer using the IVW method. (**A**) A forest plot illustrates the connections between genetically predicted factors related to overall obesity and the risk of primary mechanisms of obesity-induced cancer. (**B–I**) Scatter plots for the causal association between overall obesity and primary mechanisms of obesity-induced cancer. *BMI* body mass index, *IVW* inverse variance weighted.

The findings from the MR analysis examining the relationships between overall obesity and the primary mechanisms of obesity-induced cancer are displayed in the Table S5. IVW analysis revealed a robust positive causal relationship between BMI and circulating leptin levels (*P* = 1.23 × 10^−30^, OR = 2.489, 95% CI 2.131–2.908), C-reactive protein levels (*P* = 9.82 × 10^−54^, OR = 1.494, 95% CI 1.419–1.572), and risk of severe insulin-resistant type 2 diabetes (*P* = 5.72 × 10^−14^, OR = 3.665, 95% CI 2.612–5.143) (Fig. 3). Notably, although no significant association was observed BMI and adiponectin exhibited a positive correlation (*P* = 9.93 × 10^−01^, OR = 1.000, 95% CI 0.968–1.033) (Fig. 3). As depicted in the Fig. 3 and Table S5, IVW analysis revealed significant associations between genetically predicted body fat percentage and circulating leptin levels (*P* = 1.88 × 10^−07^, OR = 1.760, 95% CI 1.423–2.177), C-reactive protein levels (*P* = 8.47 × 10^−39^, OR = 1.903, 95% CI 1.728–2.097), risk of severe insulin-resistant type 2 diabetes mellitus (*P* = 3.11 × 10^−08^, OR = 4.091, 95% CI 2.484–6.738), and adiponectin (*P* = 1.24 × 10^−03^, OR = 1.106, 95% CI 1.040–1.176).

### Effect of primary mechanisms of obesity-induced cancer on HCC risk

To investigate the effects of primary mechanisms of obesity-induced cancer on HCC risk, we initially included a total of 263 SNPs as IVs associated with C-reactive protein levels, 6 SNPs with circulating leptin levels, 14 SNPs with adiponectin, 9 SNPs with severe insulin-resistant type 2 diabetes (Table S6). Simultaneously, the amalgamation of SNPs data from diverse primary mechanisms of obesity-induced cancer with HCC datasets is presented in Table S7.

**Figure 4:**
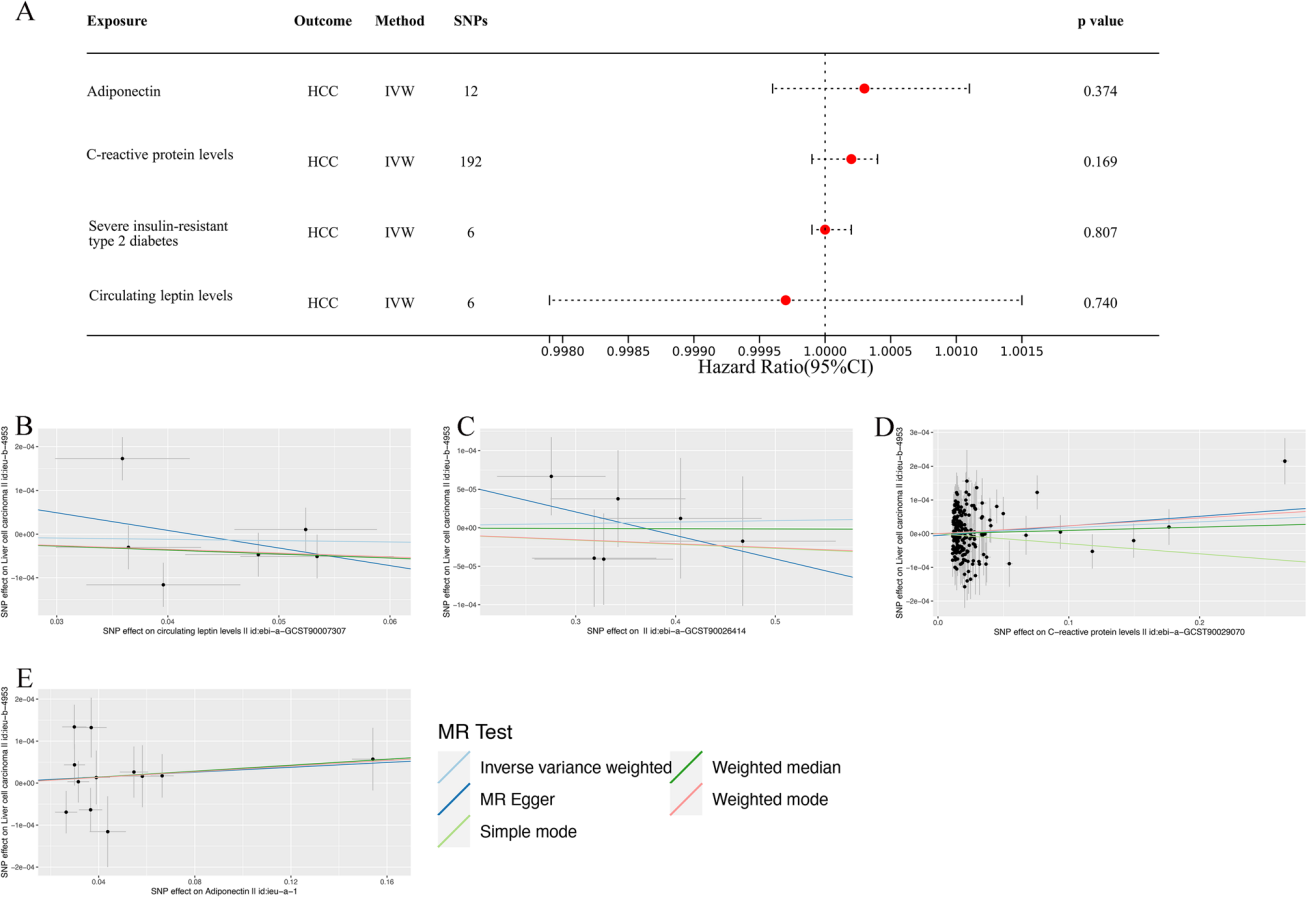
Associations of genetically predicted primary mechanisms of obesity-induced cancer (insulin resistance, adipokines—most notably, leptin and adiponectin—and chronic inflammation) with risk of HCC using the IVW method. (**A**) A forest plot illustrates the connections between genetically predicted factors related to primary mechanisms of obesity-induced cancer and the risk of HCC. (**B–E**) Scatter plots for the causal association between primary mechanisms of obesity-induced cancer and HCC. *HCC* hepatocellular carcinoma, *IVW* inverse variance weighted.

The Table S8 provides estimates from MR and power analysis comparing various primary mechanisms of obesity-induced cancer to HCC. In the IVW analysis, there is insufficient evidence to substantiate a causal connection between C-reactive protein levels (*P* = 0.169, OR = 1.0002, 95% CI 0.9999–1.0004), adiponectin (*P* = 0.374, OR = 1.0003, 95% CI 0.9996–1.0011), circulating leptin levels (*P* = 0.740, OR = 0.9997, 95% CI 0.9979–1.0015), severe insulin-resistant type 2 diabetes (*P* = 0.807, OR = 1.0000, 95% CI 0.9999–1.0002), and the development of HCC, possibly attributable to limited statistical power (Fig. 4A). Figure 4B–E displays visual scatter plots of the MR analysis results.

### Sensitivity analysis

As demonstrated in Table S9, based on the MR-Egger intercept test, all *P*-values exceeded 0.05, indicating the absence of horizontal pleiotropy. Upon conducting the Q test analysis, heterogeneity was evident among the associations involving waist-to-hip ratio, C-reactive protein levels, circulating leptin levels, and HCC, as well as BMI and C-reactive protein levels, body fat percentage and severe insulin-resistant type 2 diabetes, and adiponectin, C-reactive protein levels, circulating leptin levels (*P* < 0.05). No significant heterogeneity was observed among the remaining exposure variables and outcome variables (*P* > 0.05). The heterogeneity observed may stem from variations in the impacts of different genetic variants across distinct populations or under varying environmental conditions. Moreover, no notable outliers were identified in the MR-PRESSO or leave-one-out analyses.

### Differential expression gene analysis in HCC and obesity

**Figure 5:**
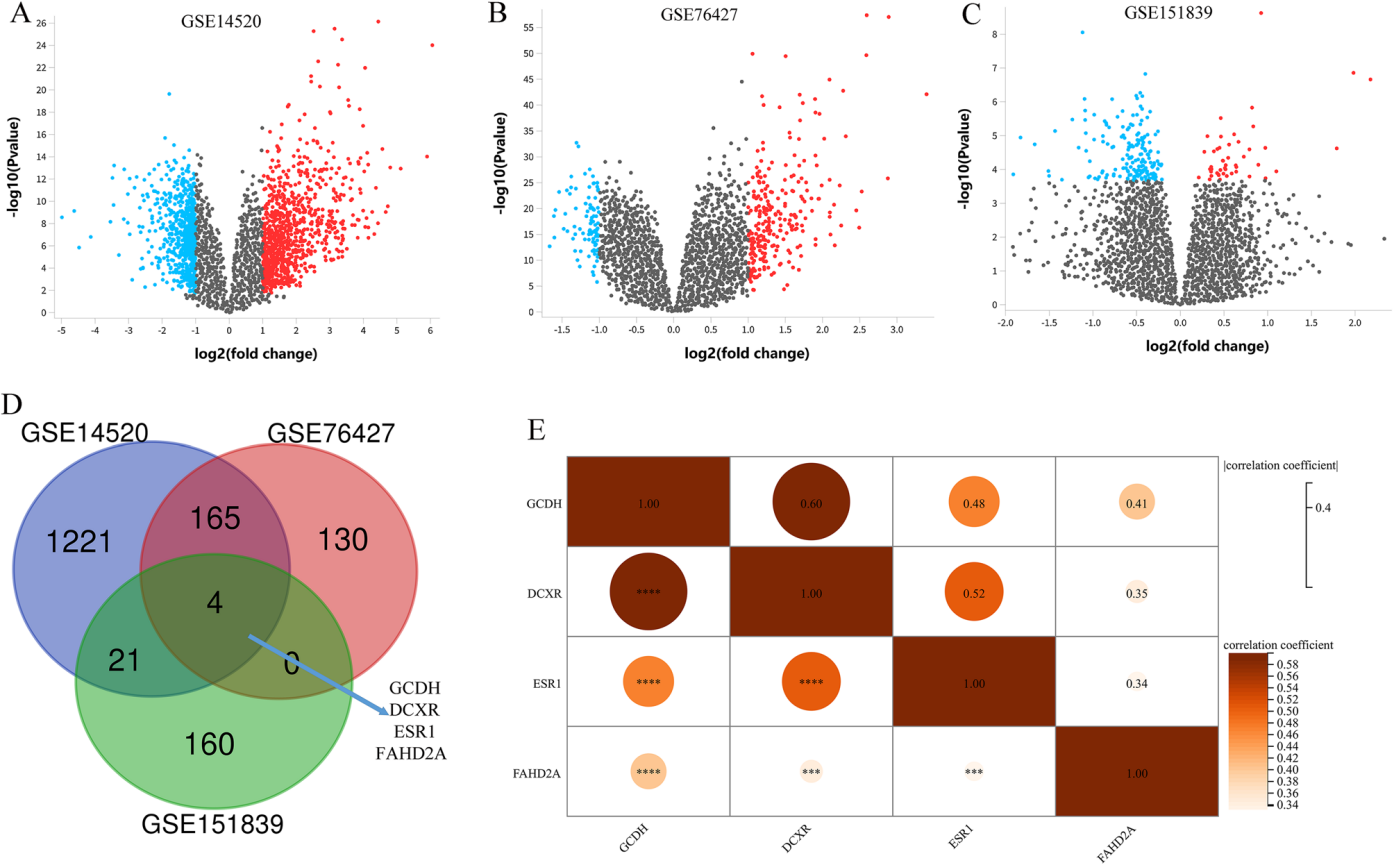
Differential expression analysis of genes in obesity and HCC datasets. (**A–C**) Volcano plot of differentially expressed genes in GSE14520 (**A**), GSE76427 (**B**), and GSE151839 (**C**). (**D**) Venn diagram of differentially expressed genes in obesity (GSE151839) and HCC (GSE14520 and GSE76427). (**E**) Co-expression analysis of four differentially expressed genes shared between obesity and HCC in HCC tissues (GSE76427). *HCC* hepatocellular carcinoma.

To identify common differentially expressed genes between HCC and obesity, we selected two HCC datasets (GSE14520 and GSE76427) and one obesity dataset (GSE151839) from the GEO database for differential expression analysis. Figure 5A–C illustrates the volcano plot’s ability to effectively represent the upregulated and downregulated genes in all three datasets. Subsequently, we employed Venn diagrams to pinpoint the common obesity-related differentially expressed genes that intersected with those from HCC and adjacent paracancerous tissues (Fig. 5D). Four differentially expressed genes, including ESR1, GCDH, FAHD2A, and DCXR, were found to overlap between HCC and obesity. To explore the functional associations of these four differentially expressed genes, we conducted co-expression analysis based on the GSE76427 dataset (Fig. 5E). The results revealed significant co-expression relationships among them, further confirming the substantial contributions of these differentially expressed genes to the development of obesity and HCC.

### PPI analysis and enrichment pathway analysis

**Figure 6:**
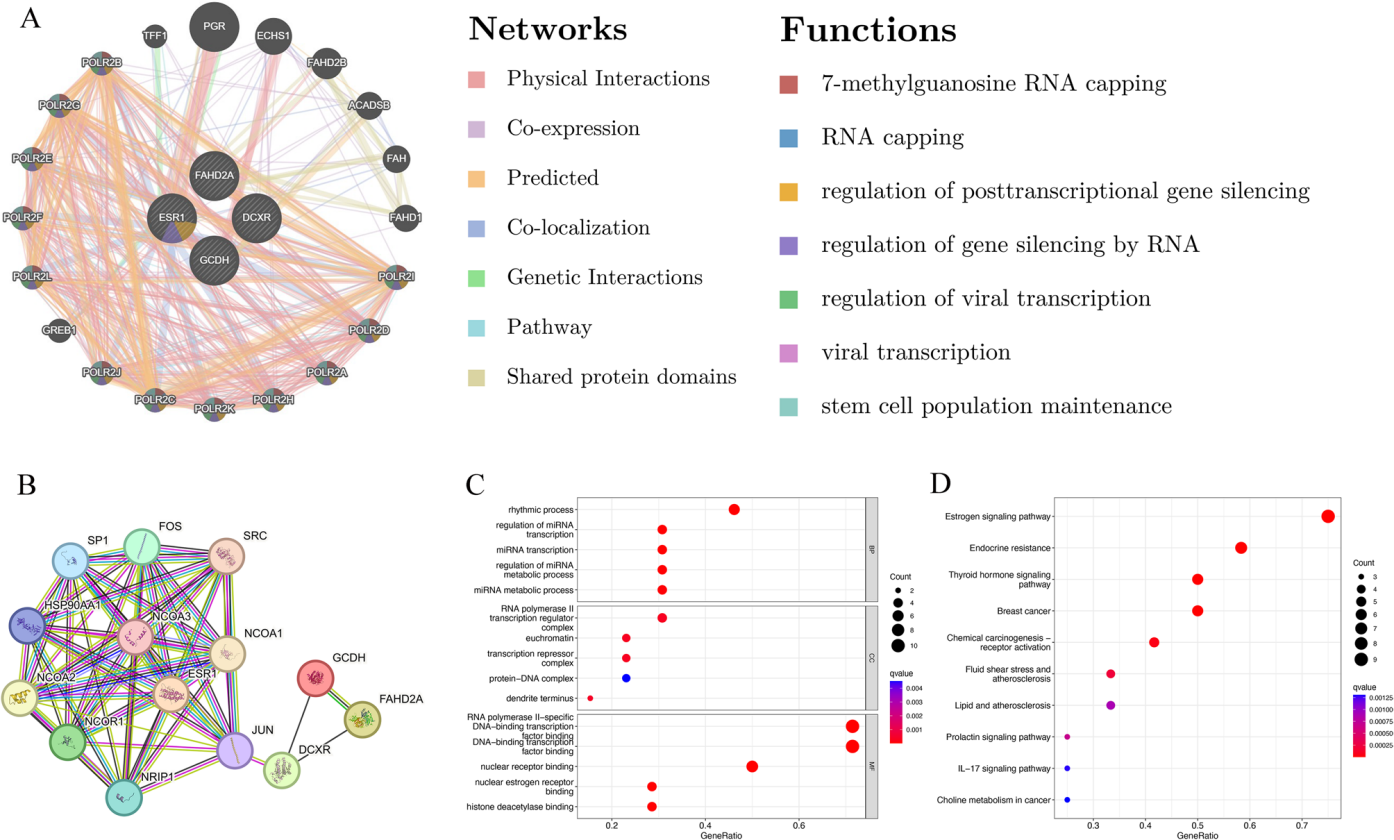
PPI and functional enrichment pathway analysis. (**A**) A PPI network of 4 differentially expressed genes using GeneMANIA. (**B**) A PPI network of 4 differentially expressed genes and 10 closely related genes using STRING. (**C**) GO enrichment analysis results of 4 differentially expressed genes and 10 closely related genes. (**D**) KEGG pathway analysis results of 4 differentially expressed genes and 10 closely related genes. *PPI* protein–protein interaction, *GO* gene ontology, *KEGG* Kyoto encyclopedia of genes and genomes.

In order to delve deeper into the biological relevance of these differentially expressed genes in hepatocellular carcinogenesis, we identified proteins and genes associated with them and proceeded with subsequent pathway enrichment analysis. We constructed the PPI network for these differentially expressed genes using GeneMANIA and STRING (Fig. 6A–B). Simultaneously, we discovered that these genes are associated with processes such as 7-methylguanosine RNA capping, RNA capping, regulation of posttranscriptional gene silencing, regulation of gene silencing by RNA, regulation of viral transcription, viral transcription, and stem cell population maintenance. Subsequently, we conducted GO and KEGG analyses on these differentially expressed genes in order to elucidate the molecular mechanisms through which obesity regulates the onset of hepatocellular carcinogenesis. As depicted in the Fig. 6C**,** the results of the GO function enrichment analysis revealed that the co-expressed differentially expressed genes were predominantly grouped within Cellular Components (CCs), specifically RNA polymerase II transcription regulator complexes and euchromatin. In terms of Biological Process (BP) functional ontologies, the co-expressed differentially expressed genes were primarily enriched in rhythmic processes and the regulation of miRNA transcription. Regarding molecular function (MF), the co-expressed differentially expressed genes were primarily associated with RNA polymerase II-specific DNA-binding transcription factor binding and DNA-binding transcription factor binding. KEGG pathway analysis revealed that these differentially expressed genes were significantly associated with choline metabolism in cancer, IL-17 signaling pathway, chemical carcinogenesis—receptor activation, endocrine resistance (Fig. 6D).

### Correlation between differentially expressed genes and the TME

**Figure 7:**
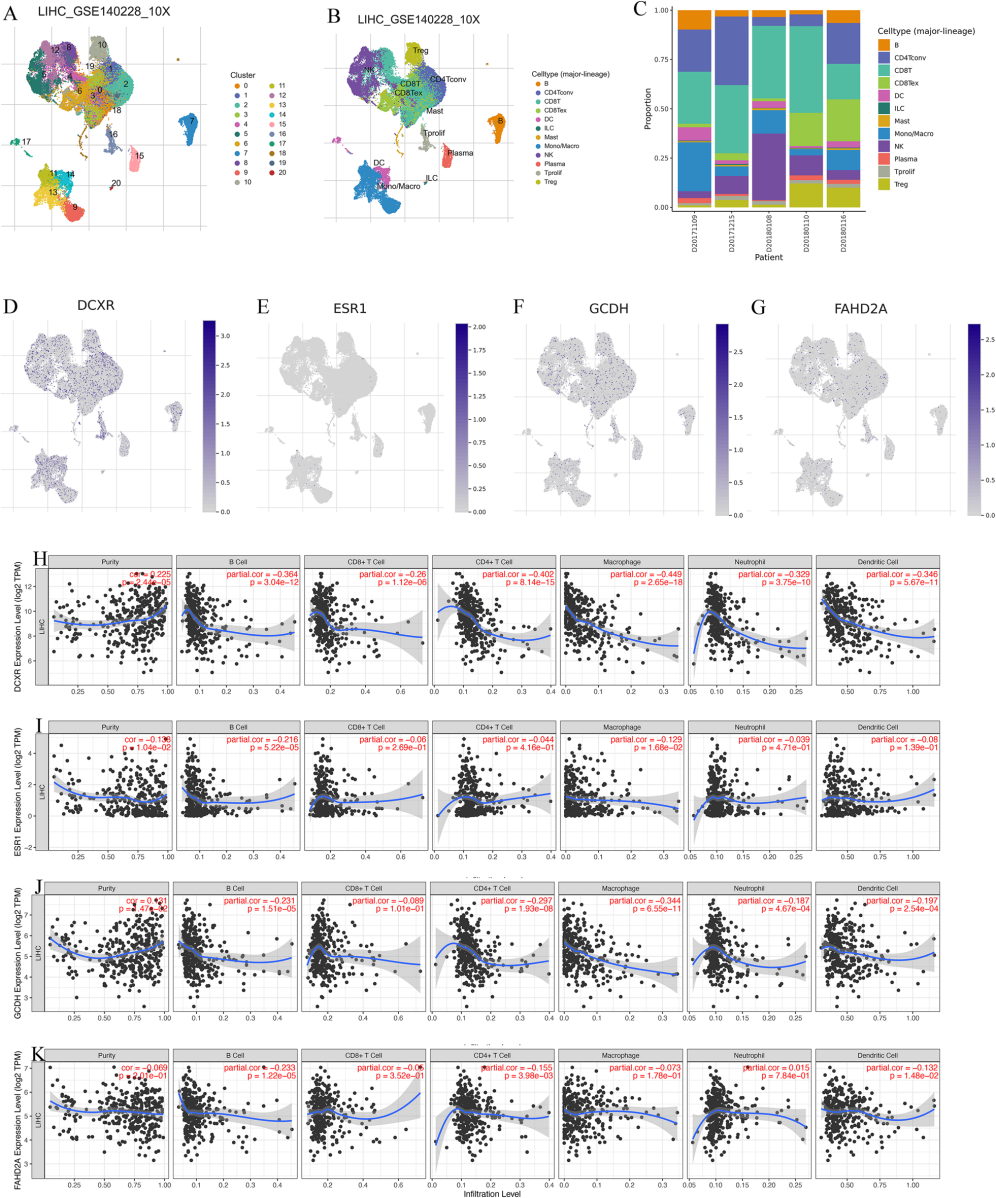
Correlation between differentially expressed genes and TME at the single-cell level. (**A–C**) Classification and statistics of cell types in the LIHC_GSE140228_10X data set. (**D–G**) Distribution and expression of the 4 differentially expressed genes (ESR1, GCDH, FAHD2A, and DCXR). (**H–K**) The correlation between 4 differentially expressed genes and immune cell infiltration in HCC. *TME* tumor microenvironment, *HCC* hepatocellular carcinoma.

We conducted an analysis of four differentially expressed genes using the LIHC_GSE140228_10X dataset available in the TISCH database, focusing on single-cell RNA sequencing data. Our analysis unveiled a total of 20 distinct cell clusters, which further aggregated into 12 average cell types (Fig. 7A–C). Our findings revealed that DCXR exhibited extensive expression in the majority of major immune cell types (Fig. 7D). In contrast, GCDH and FAHD2A displayed moderate expression across various immune cell types, while ESR1 exhibited minimal to no expression in these cell types (Fig. 7E–G). The TIMER database is used to analyze the association of four differentially expressed genes expression levels with invasive HCC immune populations (Fig. 7H–K). Interestingly, DCXR exhibited negative correlations with the infiltration of B cells, CD8 + T cells, CD4 + T cells, macrophages, neutrophils, and dendritic cells. ESR1 displayed negative correlations with the infiltration of B cells and macrophages. GCDH showed negative correlations with the infiltration of B cells, CD4 + T cells, macrophages, neutrophils, and dendritic cells. Meanwhile, FAHD2A exhibited negative correlations with the infiltration of B cells, CD4 + T cells, and dendritic cells.

### Analysis of immunotherapy relevance and drug sensitivity

**Figure 8:**
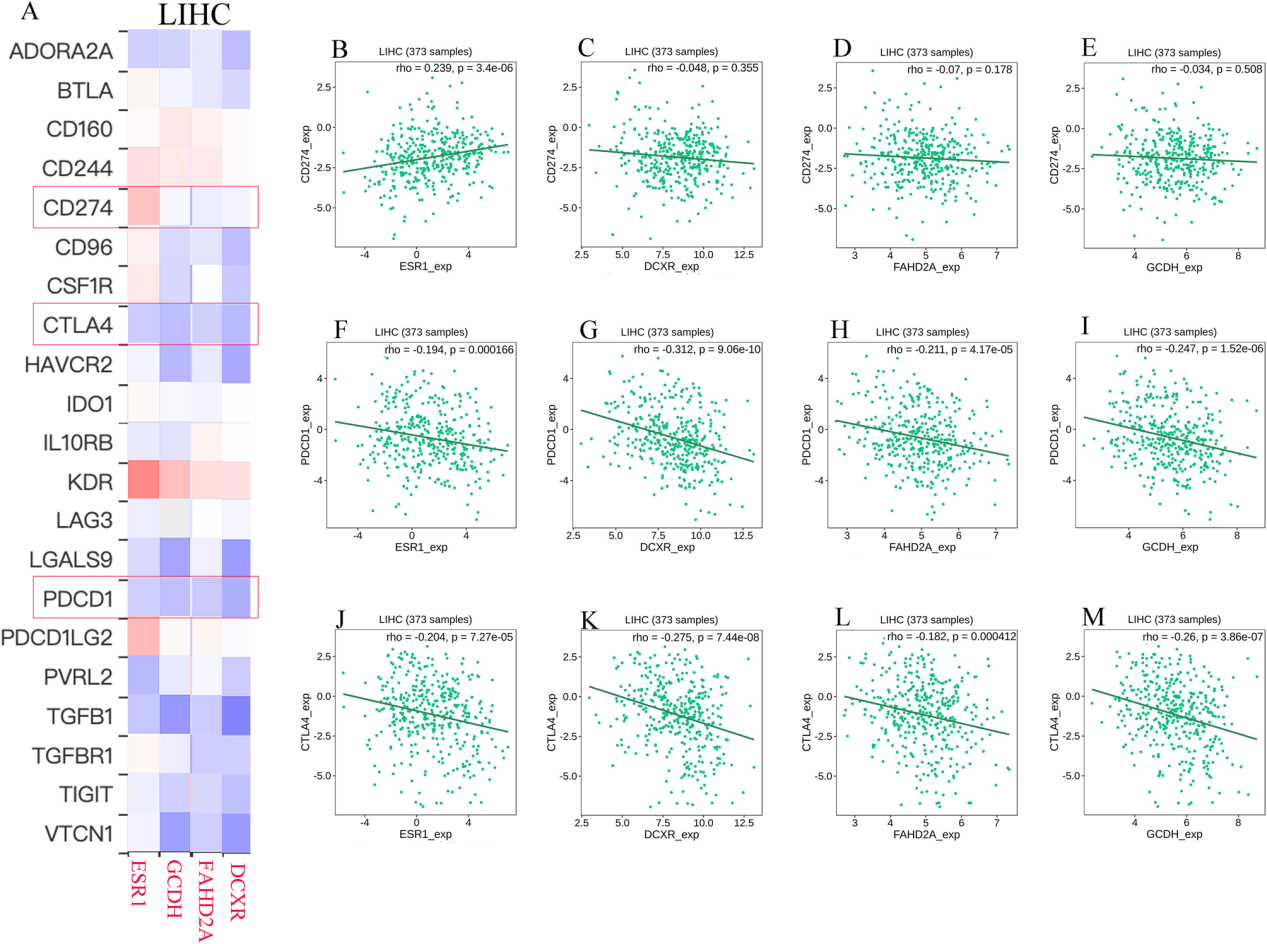
Correlation between differentially expressed genes and Immune checkpoint inhibitors in HCC. (**A**) A heatmap displays the correlation between 4 differentially expressed genes and immune checkpoint inhibitors in HCC. (**B–M**) A scatter plot illustrates the correlation between the expression of 4 differentially expressed genes and PD-1, PD-L1, and CTLA-4. *HCC* hepatocellular carcinoma.

**Figure 9:**
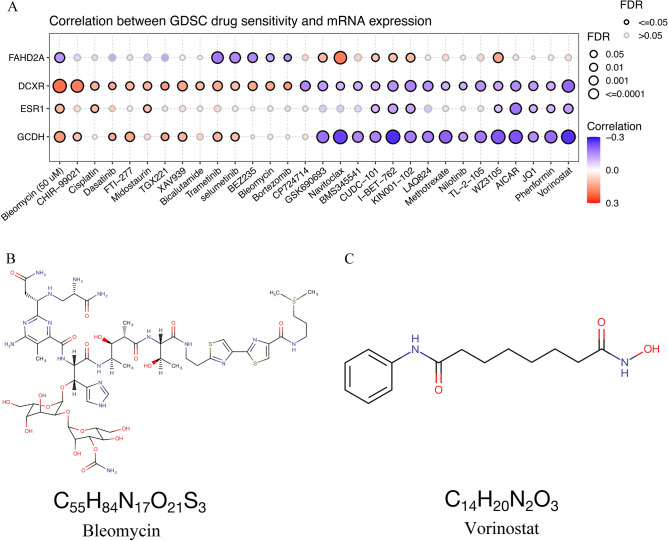
Gene-drug interaction and drug sensitivity analysis. (**A**) The connection between the expression of 4 differentially expressed genes and drug responsiveness in the GSCA database. (**B–C**) The molecular formulas of Bleomycin and Vorinostat.

Immune checkpoint inhibitors are the primary approach in current tumor immunotherapy. Results from the TISIDB database indicate that four differentially expressed genes are negatively correlated with the expression of most immune checkpoints (Fig. 8A). PD-1, PD-L1, and CTLA4 are among the most commonly used immune checkpoints in clinical practice. Our results indicate that, except for the positive correlation between ESR1 and CD274 (R = 0.235, *P* = 3.4e − 06), all others exhibit a negative correlation (It should be noted that FAHD2A and GCDH show no significant correlation with CD274) (Fig. 8B–M). Additionally, we performed expression-related correlation analysis on the four differentially expressed genes using the GDSC drug sensitivity dataset from the GSCA database (Fig. 9A). We observed a significant positive correlation between DCXR expression and sensitivity to Bleomycin. Conversely, GCDH expression exhibited a significant negative correlation with Vorinostat sensitivity. To gain a better understanding of these two drugs, we have provided their molecular formulas below (Fig. 9B–C).

## Discussion

Obesity is a prominent global health concern that directly impacts liver health, leading individuals with obesity to face a substantial risk of elevated tumorigenesis in the liver^13,21^. Obesity, arising from an aberrant metabolic milieu, leads to modifications in immune function, initially manifesting as a state of persistent low-grade systemic inflammation and, in some cases, progressing to immune dysfunction^23,24^. Obesity represents a standalone risk factor for numerous cancer types, including HCC^25^. HCC stands as the most common primary liver malignancy and ranks among the top causes of cancer-related fatalities worldwide^10,26^. Prior studies have demonstrated that the risk of HCC increases by 17% in overweight individuals and rises to 89% in those with obesity, underscoring the association between the severity of metabolic imbalances and liver pathology^27,28^. Various mechanisms have been identified that potentially connect obesity to the development of cancers like HCC. These mechanisms include abnormal hormone and cytokine activity, exposure to metabolic toxins, oxidative damage, disruptions in the cell cycle, and compromised immune function^29–32^. Obesity and the primary mechanisms underlying obesity-related traits have been identified as potential factors associated with an elevated risk of HCC in various observational studies^33,34^. Nevertheless, a comprehensive report on the association between obesity and hepatocellular carcinogenesis is lacking, and the relationship with HCC has yet to be explored within the broader context of obesity as a whole. Furthermore, it is essential to acknowledge that observational studies may not definitively establish causality, as residual confounding and reverse causality are potential limitations. Accordingly, our research initiated a two-sample MR study as the primary approach to investigate the potential causal association between obesity and the susceptibility to HCC.

Obesity serves as a risk factor for various cancers, including HCC^13,35,36^. In this current study, we conducted two-sample MR analyses to explore potential causal links between different obesity-related traits and the risk of HCC in the European population. Our investigation revealed that among two indicators of overall obesity, genetically determined BMI and body fat percentage, as opposed to waist circumference, waist-to-hip ratio, and visceral adipose tissue volume (reflecting specific fat distribution), displayed significant associations with HCC. These findings imply that overall obesity plays a pivotal role in the development of HCC. In addition to the lack of causal relationship found between BMI and adiponectin, our two-sample MR analysis revealed a significant causal relationship between overall obesity and the primary mechanisms of obesity-induced cancer, which include insulin resistance, adipokines, and chronic inflammation. These findings suggest that obesity may increase the risk of cancer by triggering a cascade of biologic mechanisms associated with cancer. To gain deeper insights into whether obesity contributes to the development of HCC through these shared biological mechanisms, we performed causal association analyses between these mechanisms and HCC. Regrettably, our study did not find significant causal associations between primary mechanisms of obesity-related cancer and HCC risk, highlighting potential challenges in detecting subtle genetic effects and the complexity of underlying biological interactions. Limited statistical power, due to small sample sizes and genetic variability across populations, likely contributed to these findings. The results emphasize the importance of using larger, multi-ethnic cohorts and advanced statistical models to fully capture the intricate relationships between obesity and HCC. These insights underline the necessity for a multifactorial approach in clinical practice that considers both genetic and environmental factors. Overall, our findings encourage a broader exploration of obesity’s role in HCC pathogenesis, stressing the need for comprehensive studies that integrate diverse data sources and complex biological frameworks.

Our findings highlight the significant implications of the causal link between obesity and increased risk of HCC for clinical practices. Specifically, they suggest the integration of more aggressive obesity management strategies and tailored HCC screening protocols, especially for genetically predisposed populations. These adjustments could potentially enhance early detection and improve preventive measures against HCC in obese individuals, ultimately leading to better health outcomes.

In past research, the formation of chronic liver inflammation can lead to hepatocyte damage and oxidative stress through various mechanisms^21,37^. Chronic oxidative stress promotes DNA damage and compensatory repair processes, thereby promoting gene mutation and tumorigenesis^38^. An overaccumulation of fat within adipose tissue can trigger an increase in the expression of pro-inflammatory adipokines while concurrently decreasing the expression of anti-inflammatory adipokines, which ultimately contributes to the establishment of chronic inflammation^39^. Insulin resistance and the ensuing hyperinsulinemia stand as significant pathological outcomes of obesity, and these conditions have also been identified as contributing factors in the development of HCC^40^. Insulin resistance leads to increased serum levels of insulin-like growth factor 1 (IGF-1) and enhances the biological activity of IGF-1. Both IR and IGF-1 bind to the insulin-like growth factor 1 receptor (IGF-1R), which activates downstream cellular pathways, such as phosphatidylinositol-3 kinase (PI3K). This activation induces proliferation and inhibits apoptosis in HCC cells, ultimately promoting the tumorigenesis of HCC^41^.

Tumor development can result from the inactivation of tumor suppressor genes and the overexpression of oncogenes^4^. In the present study, we have identified four differentially expressed genes shared between obesity and HCC, namely ESR1, GCDH, FAHD2A, and DCXR. These genes may play a pivotal role in exploring the mechanisms underlying obesity-induced HCC. Through PPI enrichment analysis, we found that these genes play a role in cell cycle processes, including 7-methylguanosine RNA capping, RNA capping, regulation of posttranscriptional gene silencing, and regulation of gene silencing by RNA. Simultaneously, we observed that differential gene expression also affects the regulation of viral transcription. Migdal et al.^42^ similarly found that obesity can accelerate the progression of liver fibrosis in patients with hepatitis C virus infection. Previous studies have also confirmed that the risk of HCC significantly escalates when hepatitis C virus infection patients concurrently experience alcoholism or obesity, with the odds ratio increasing from 8–12 to 48–54^43–45^. Therefore, we hypothesize that differentially expressed genes within the context of obesity may accelerate the progression of viral hepatitis-induced liver fibrosis and cirrhosis by regulating cellular processes, ultimately leading to the development of HCC. The KEGG analysis also revealed that differentially expressed genes are associated with the IL-17 signaling pathway, which is involved in inflammation, as well as endocrine resistance. This evidence collectively reaffirms the close relationship between obesity in patients and the development of HCC.

The development and sustenance of cancer hallmarks, including the promotion of cell proliferation, resistance to cell death, initiation of angiogenesis, facilitation of invasion and metastasis, induction of tumor-promoting inflammation, and evasion of immune responses, are influenced to varying degrees by the contributions from the TME^46,47^. The composition of immune cells infiltrating the TME are of paramount importance regarding tumor progression^48^. Our research has revealed a negative correlation between differential gene expression and immune cell infiltration in HCC. This suggests that elevated expression of these differentially expressed genes is linked to decreased immune cell infiltration in the TME, resulting in an immunologically cold tumor. Simultaneously, we have observed a predominantly negative correlation between the expression levels of these differentially expressed genes and common immune checkpoint markers, which may imply reduced responsiveness to immune checkpoint inhibitors in obese patients. This hypothesis requires further validation with additional clinical data.

This study has certain limitations to be considered. Firstly, our study data were sourced from various databases, including the MRC-IEU consortium, Neale Lab, GIANT, UK Biobank, GIANT, and FinnGen consortium, potentially introducing selection bias. Moreover, these databases predominantly comprise European populations, which limits the ability to establish a causal relationship between obesity and HCC risk. Further investigations are necessary to determine the generalizability of our findings to other populations. Additionally, it’s worth noting that there has been insufficient research into the molecular mechanisms and functions underlying obesity-induced HCC formation.

## Conclusion

In conclusion, our study provides evidence supporting a causal relationship between overall obesity and an increased risk of HCC. Although no significant causal associations were detected between the primary mechanisms of obesity-related cancer and HCC risk, this may be attributed to limited statistical power. Furthermore, we have identified four genes that exhibit differential expression in both obesity and HCC, implicating their involvement in the development of obesity-induced HCC. Nevertheless, further research is required to fully elucidate the precise molecular mechanisms involved.

### Supplementary Information

Supplementary Tables.

## Data Availability

The datasets used and analyzed in the present study are available from the corresponding authors on reasonable request. The datasets generated and/or analyzed during the current study are available in GWAS (https://gwas.mrcieu.ac.uk/) database.

## Abbreviations

HCC: Hepatocellular carcinoma
SNPs: Single-nucleotide polymorphisms
MR: Mendelian randomization
IVW: Inverse variance weighted
MRE: MR egger
WMed: Weighted median
WMod: Weighted mode
SMod: Simple mode
CI: Confident interval
IVs: Instrumental variables LD linkage disequilibrium
KEGG: Kyoto encyclopedia of genes and genomes
PPI: Protein–protein interaction
TME: Tumor microenvironment
BMI: Body mass index
GWAS: Genome wide association study
